# Pscroph, a parasitic plant EST database enriched for parasite associated transcripts

**DOI:** 10.1186/1471-2229-5-24

**Published:** 2005-11-16

**Authors:** Manuel J Torres, Alexey A Tomilov, Natalya Tomilova, Russell L Reagan, John I Yoder

**Affiliations:** 1 Department of Plant Sciences, University of California, Davis, Davis, CA 95616, USA; 2 Plant Genome Mapping Laboratory, Center for Applied Genetic Technologies, 111 Riverbend Road #224, University of Georgia, Athens, Georgia 30602, USA; 3 Vavilov Institute of General Genetics, Russian Academy of Sciences, Gubkina Str., 3, Moscow, Russia

## Abstract

**Background:**

Parasitic plants in the Orobanchaceae develop invasive root haustoria upon contact with host roots or root factors. The development of haustoria can be visually monitored and is rapid, highly synchronous, and strongly dependent on host factor exposure; therefore it provides a tractable system for studying chemical communications between roots of different plants.

**Description:**

*Triphysaria *is a facultative parasitic plant that initiates haustorium development within minutes after contact with host plant roots, root exudates, or purified haustorium-inducing phenolics. In order to identify genes associated with host root identification and early haustorium development, we sequenced suppression subtractive libraries (SSH) enriched for transcripts regulated in *Triphysaria *roots within five hours of exposure to *Arabidopsis *roots or the purified haustorium-inducing factor 2,6 dimethoxybenzoquinone. The sequences of over nine thousand ESTs from three SSH libraries and their subsequent assemblies are available at the Pscroph database . The web site also provides BLAST functions and allows keyword searches of functional annotations.

**Conclusion:**

Libraries prepared from *Triphysaria *roots treated with host roots or haustorium inducing factors were enriched for transcripts predicted to function in stress responses, electron transport or protein metabolism. In addition to parasitic plant investigations, the Pscroph database provides a useful resource for investigations in rhizosphere interactions, chemical signaling between organisms, and plant development and evolution.

## Background

Parasitic plants directly invade and rob nutrients from host plants [1,2]. The consequences can be devastating to the host plant and some of the world's most pernicious agricultural pests are parasitic weeds [3]. The number of parasitic angiosperms is surprisingly large with over four thousand parasitic species identified in nineteen different plant families [4]. Parasitic plants have a wide diversity of growth habits ranging from the tiny flowered mistletoes that live in the tops of trees to the enormously flowered and rootless *Rafflesia *whose entire vegetative body is endophytic [4]. The degree to which parasites rely on host resources also varies. Some obligate parasites, like *Rafflesia*, have lost photosynthetic capabilities and are fully heterotrophic. Others, like Triphysaria, are facultative parasites that can mature without a host plant but will parasitize neighboring plants when available.

The single feature shared by all parasitic plants is the ability to invade host tissues via a haustorium [1]. Haustoria of parasitic plants fulfill multiple functions including host attachment, penetration, and translocation of resources from host to parasite [5]. Interestingly, the competence to develop haustoria has originated in autotrophic ancestors multiple times during the evolution of angiosperms [6]. There are two general hypotheses for the evolutionary origins of haustoria. One hypothesis suggests that the genes encoding haustorium development are derived from non-plant organisms, such as bacteria or fungi, that are endophytic or which have transferred a set of genes required for haustorium formation into the parasite genome [7]. The second is that genes encoding haustorium development are derived from those present in autotrophic angiosperms where they fulfill functions unrelated to parasitism. The identification of genes associated with haustorium development will provide insights into the evolutionary origins of plant parasitism. These genes will also elucidate the degree to which haustoria in different parasitic families are encoded by convergent or homologous genetic pathways.

Parasitic plants in the Orobanchaceae develop haustoria on their roots in response to contact with host roots. Several molecules, typically products of the phenylpropanoid pathway, have been identified that induce haustorium development when applied to Orobanchaceae roots *in vitro *[5,8-10]. Early haustorium development in response to exogenous signal molecules is characterized by three visible phenotypes: temporary cessation in root elongation, isodiametric cortical swelling, and haustorial hair proliferation [11,12].

Molecular phylogeny places the Orobanchaceae on a single phylogenetic clade of parasites distinct from the nearest non-parasitic relative [13]. This suggests that the genetic mechanisms controlling haustorium development in the Orobanchaceae are likely similar. *Triphysaria*, formerly *Orthocarpus*, is an Orobanchaceae that grows as a common, springtime annual throughout the Pacific coast from Canada to Baja [14]. *Triphysaria *is a small genus of five intercrossing diploid species that are amenable to classical genetic analyses [15]. *Triphysaria *is closely related to the devastating agricultural weeds *Striga *and *Orobanche*; however, *Triphysaria *itself has no agricultural significance. *Triphysaria *are facultative parasites that can grow to maturity without host plants but will readily parasitize many host species when available, including *Arabidopsis *and maize. *Triphysaria *form haustoria within twelve hours of being exposed to *Arabidopsis *roots or root factors *in vitro *[16]. The speed, synchrony, and dependence on exogenous inducer makes haustorium development in Orobanchaceae an excellent system for identifying transcripts associated with subterranean plant-plant communications.

Towards the goal of identifying genes associated with plant parasitism, we sequenced cDNA libraries enriched by suppression subtractive hybridization (SSH) [17] for transcripts regulated in *Triphysaria *roots during haustorium development. To date we have sequenced approximately nine thousand ESTs from three SSH libraries generated after treating *Triphysaria *roots with either intact *Arabidopsis *roots or the chemical haustorium inducer 2,6-dimethoxybenzoquinone (DMBQ). DMBQ, first purified as a haustorium inducer from sorghum [9], induces high rates of haustorium development in *Triphysaria*; however its role in mediating haustorium formation in *Triphysaria *– *Arabidopsis *interactions is not known. The Pscroph database provides on-line access to these EST and assembly sequences and provides BLAST and keyword search functions [18]. Comparative analysis with other transcriptomes will highlight genes and pathways associated with the origins of haustorium development and the evolution of heterotrophy in plants. These studies may provide insights into genetic strategies for developing crops resistant to parasitic weeds and into strategies for exploiting allelopathic interactions in agriculture generally.

## Construction and content

### Parasite treatments

*Triphysaria versicolor *seeds were collected from thousands of cross-pollinating plants growing in a grassland stand near Napa CA. They were surface sterilized in a solution of 2% sodium hypochlorite (50% household bleach) and 0.01% Triton X-1000, rinsed thoroughly with water and germinated at 16°C in 0.25X Hoagland's solution and 1% agar [19]. After two to three weeks the seedlings were transferred along one edge of a square Petri dish containing the appropriate media and incubated at 25°C at a near vertical angle so that the roots grew down along the surface of the agar. *Arabidopsis *Columbia seeds were obtained from Lehle seeds (Round Rock, TX, USA), surface sterilized, and germinated in agar. *Arabidopsis *seedlings were then cultivated hydroponically for 40 days in liquid media (30 seedlings in 30 ml of 0.25X Hoagland's media in 250 ml flasks shaking at 50 rpm at 25°C under a 16 hours light/8 hours dark cycle).

**Figure 1:**
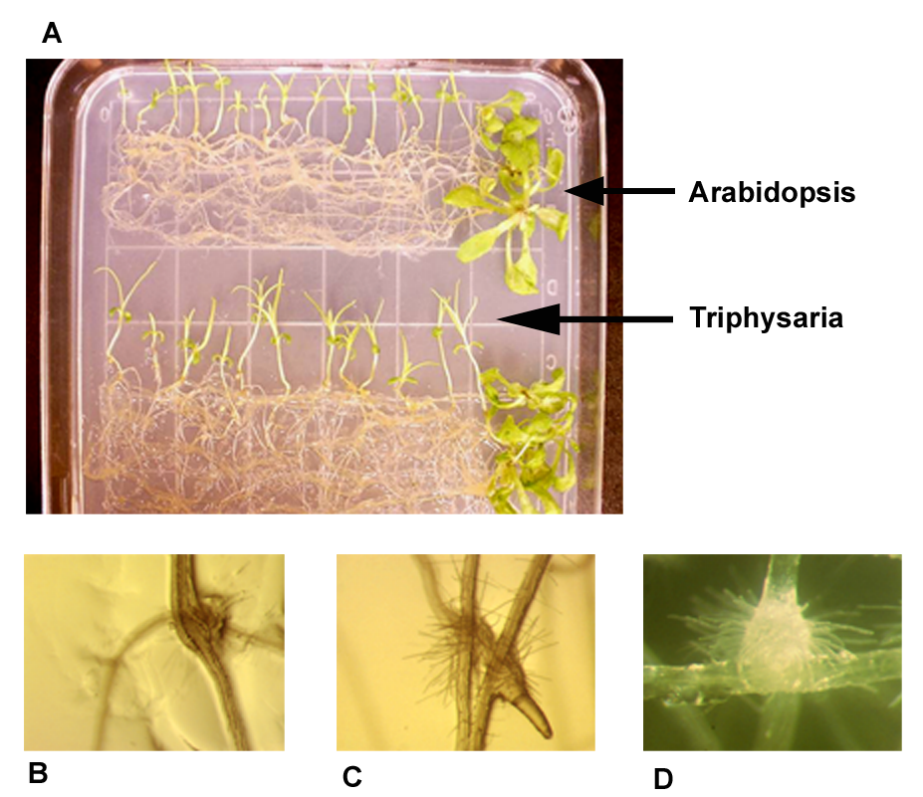
**Photos of *Triphysaria *haustoria with *Arabidopsis *host roots**. A. Physical contact between parasite and host roots was made by laying *Arabidopsis *seedlings across the roots of *T. versicolor *seedlings in vitro. RNA for the host-induced library was isolated from the *Triphysaria *roots up to five hours after contact with *Arabidopsis*. B and C. Haustorium development on *Triphysaria *roots after 36 hours contact with *Arabidopsis*. C shows a single *Triphysaria *root forming haustoria on two different *Arabidopsis *roots. D. Haustorium development after 24 hrs exposure to 30 μM DMBQ.

Haustorium development was induced five to seven days after the transfer of *Triphysaria *seedlings to vertical square plates by exposing their roots to *Arabidopsis*. This was done by placing roots of forty day old hydroponically grown *Arabidopsis *across the roots of *Triphysaria *seedlings as shown in figure 1a. Two ml of 0.25X Hoagland's media were applied to the roots to ensure good contact. Contact was maintained for up to five hours during which time *Triphysaria *roots were harvested for RNA. Haustoria were also induced by applying two ml of 10 μM DMBQ directly to *Triphysaria *roots for construction of the EDIT library.

Early haustorium development could be detected within 24 hr of host parasite contact as swollen, hairy knobs emerging just proximal to the *Triphysaria *root tips (Figure 1b,c,d). *Triphysaria *exposed to media without *Arabidopsis *or DMBQ did not develop haustoria.

### Library construction

*Triphysaria *roots were dissected and frozen in liquid nitrogen at various time intervals ranging from immediately after treatment to up to five hours later. *Triphysaria *RNA was prepared as described [20]. Suppressive subtractive hybridization was used to generate cDNA libraries enriched for up or down regulated transcripts using a commercial kit (BD Sciences, Clontech, Mountain View, CA). The Host Forward (HF) library was enriched for transcripts upregulated in *Triphysaria *roots after contact with *Arabidopsis *and was made using mock-treated *Triphysaria *as the hybridization driver. The Host Reverse (HR) library was enriched for transcripts down regulated after host contact and was made using *Arabidopsis *exposed *Triphysaria *as driver. The EDIT library was enriched for transcripts upregulated in root tips within five hours of exposure to DMBQ as previously described [20].

### EST sequencing and assembly

Subtracted cDNAs were ligated into pCR2.1-Topo (Invitrogen, Carlsbad, CA) and cloned in *E. coli*. Colonies were picked into 384 well microtiter plates and frozen at -80°C. Sequencing templates were prepared from the library using a rolling circle amplification technology with TempliPhi kit (Amersham Biosciences, Piscataway, NJ). Sequencing reactions were run using the BigDye terminator v3.1 (Applied Biosystems, Foster City, CA.) and the products were separated and detected using the ABI3730xl DNA Fragment Analyzer (Applied Biosystems, Foster City, Foster City, CA) at the UC Davis College of Agriculture and Environmental Sciences Genomics Facility.

DNA trace files were base-called using Phred (version 0.990722.g) and low quality sequences were removed based on a Phred p value ≤ 0.05 [21]. The sequences were masked for the pCR 2.1-TOPO cloning vector, linker sequences, and repetitive sequences (excluding poly A and poly T) based on alignments generated by the BLASTN program as used by the PyMood Sequence Processor (Allometra, Davis, CA) (Alexander Kozik, pers. comm.). Sequences less than 100 nts were discarded from further analyses. Approximately five percent of the clones had linker sequences internal to an ORF sequence. These were determined to be chimeras generated by the ligation of multiple SSH fragments into a single plasmid. The chimeric sequences were computationally digested into independent ESTs. The finished ESTs were submitted to GenBank's dbEST repository [22].

FASTA files of the finished ESTs were assembled into contigs using the cap3 program. Because we assembled trimmed and masked FASTA sequences, quality files were not included and the cap3 clipping function was unnecessary. The assembly was performed at the default parameters (overlap length cutoff = 30; overlap percent identify cutoff = 75; and overlap similarity score cutoff = 500) [23]. Fifty percent of the assemblies were comprised of a single EST; an additional forty percent were comprised of two, three, or four ESTs. The assembly process identified about 1100 transcript assemblies in the HF library, 1300 in the HR library and 1400 in the EDIT library (Table 1).

### Database of early haustorial transcripts

The EST sequences and assembly alignments are available at the Pscroph database [18]. Data are stored in a MySQL database and made available on the web using a phpMyAdmin interface. The database is housed at the University of California-Davis Genome Center.

Proteins predicted to be encoded by the assemblies were annotated from the BLASTX reports comparing *Triphysaria *sequences to either all proteins in GenBank (rel145.fsa_aa release Dec 15, 2004) or to all predicted proteins in *Arabidopsis *(ATH1.pep_cm_20040228). These BLAST reports can be accessed at the web site as full text files or by keyword searches of protein annotations. The keyword search function reports the best three hits obtained from GenBank or TAIR databases with e values ≤ 10^-8^. Each best hit is hyperlinked to the corresponding report page at NCBI or TAIR. The web site also provides a BLAST function that allows homology searches against DNA or protein sequences in each or all libraries.

## Utility and discussion

### SSH libraries

We previously published a sequence characterization of 246 cDNAs from the EDIT library [20]. At that time we sequenced clones that had been selected by colony hybridizations for transcripts most differentially abundant in the forward hybridization reaction compared to the reverse. This is suggested by the manufacturer to reduce the number of false positives. The colonies not analyzed fell into two, roughly equal sized groups; those that hybridized to both forward and reverse probes and those that hybridized to neither. While this step reduces the number of false positives, it also may eliminate interesting transcripts. In particular, colonies that hybridized with neither probe likely represented weakly expressed, low abundance transcripts [24]. Furthermore, colonies that hybridized to probes from multiple libraries may contain conserved domains in otherwise distinct proteins. Therefore we sequenced additional, unselected clones from the EDIT library and eliminated the hybridization in constructing the HF and HR libraries.

**Figure 2:**
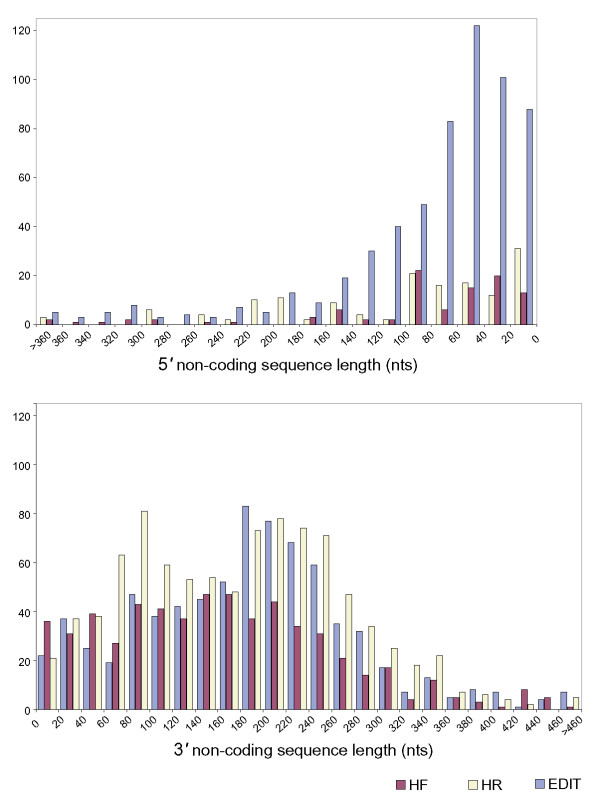
**Length of 5' and 3' non-coding sequences**. Protein translations of the SSH sequences in each library were mapped by BLASTX to the most homologous plant protein in GenBank. The histogram depicts the number and size of SSH sequences predicted to extend into either the 5' or 3' non-coding regions.

The SSH procedure included an *Rsa*1 digestion step prior to cloning that resulted in bidirectional cloning and in single transcripts being represented by multiple, non-overlapping SSH products. In order to determine the distribution of SSH products relative to the 3' and 5' ends of the encoding gene, we mapped the virtual translations of the SSH ESTs onto the most homologous protein in the plant protein database. The tcl_blast_parser_123_V017 was used to convert BLASTX output data to a table format suitable for manipulation in a spreadsheet [25]. Using the length of the target ORF and the amino acid locations corresponding to the start and stop of the aligned region between the SSH and plant homologs, we estimated the number and length of *Triphysaria *sequences predicted to be either 3' or 5' non-coding (Table 2, Figure 2). These regions provide good candidate sites for identifying gene specific primers.

Depending on the library, from 34 % to 62% of the *Triphysaria *sequences were predicted to include non-coding sequences; one to ten percent of the cDNAs included both 5' and 3' non-coding sequences (Table 2). There were more 3' than 5' non-coding sequences in all libraries; there were eight times more 3' sequences in the HR library. The 3' non coding regions recovered in the SSH libraries were also longer than those predicted for the 5' (Figure 2). The 3' bias likely results from the initial cDNA synthesis reaction that is primed with poly-T. Depending on the library, between ten and twenty percent of the SSH products had poly-A tracts. The predominance of ORF encoded sequences in the libraries demonstrates that these libraries were less biased towards 3' sequences than would be expected without the *Rsa*1 digestion.

### Interlibrary comparisons

**Figure 3:**
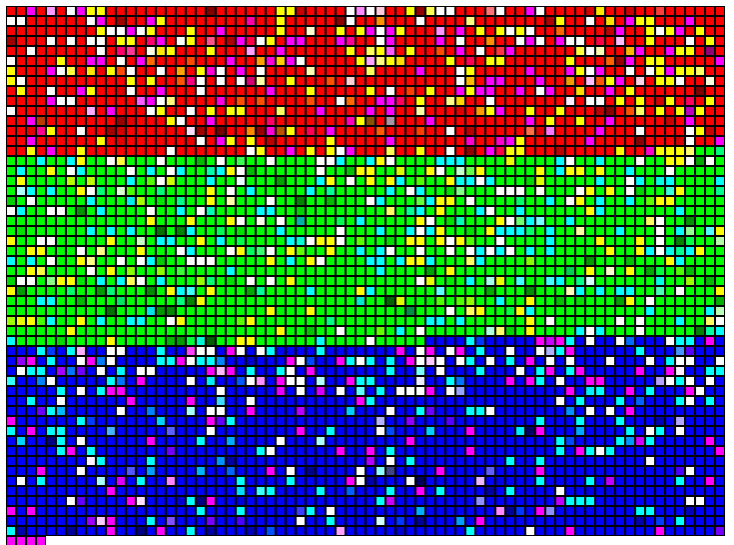
**Virtual cDNA arrays and clone redundancy in different libraries**. A FASTA file containing all HF, HR and EDIT assembly sequences was used as the target in BLASTN comparisons with sequences from each library as query. Each target cDNA (3820 total) was assigned a color based on homology to sequences in different libraries; sequences hybridizing to HF probes were assigned red, those hybridizing to HR probes green, and those with EDIT probes blue. The color intensity reflected the BLASTN score with higher values assigned to greater homology. Colors were mixed when sequences were present in more than one library: those present in both the HF and HR libraries were yellow, in both the HF and EDIT libraries pink, and in the HR and EDIT libraries teal. Assembly sequences with homologies in all three libraries are represented as white.

We used BLASTN to identify nucleotide sequences in common between the different libraries. This is a bioinformatics alternative to colony hybridization to identify interlibrary sequence homologies. Figure 3 is a color representation of the BLASTN results generated by the PyMood software package (Allometra, Davis, CA). The squares represent 3820 assembly sequences arrayed in the order HF, HR and EDIT. BLASTN was performed using the concatenated sequences from the virtual array as target and sequences from each library as query. PyMood parsed the BLAST output and assigned mixes of red, blue and yellow colors to each sequence based on the degree to which the target sequence had homologies in other libraries. The intensity of color was a function of the BLASTN e value and colors were mixed when sequences were present in more than one library.

As shown in table 3, about seventy percent of the sequences were specific to a single library. About seven percent of the assemblies were found in both HF and EDIT libraries but not HR; these represent likely candidates for early haustorium development. However similar numbers of sequences were in common between the HF and HR libraries, indicating a basal level of interlibrary redundancy. The number of sequences in common between forward and subtracted libraries is higher than expected if there was no selection for particular cross-hybridizing sequences. If the one thousand sequenced assemblies in each library represent 2% of the approximately 20,000 root transcripts [26], about 0.4% of assemblies would be expected in both libraries by chance alone. In a previously published wet lab characterization of the EDIT library, we reported that about 20% of the clones cross-hybridized to transcripts in both forward and reverse subtracted probes. Other experiments employing SSH procedures report false positive rates of cross-hybridizing clones of 30–50% [27,28]. The Clonetech Selelect PCR users guide states that recovery rates of false positives will vary between tissue types and RNA preparations [29].

The unpredictably high rate of cross-library hybridizing transcripts was not a function of the assembly because a BLASTN analysis of EST sequences before assembly gave similar results (data not shown). Approximately half of the cross-hybridizing sequences had multiple sequence polymorphisms, suggesting these are alleles of co-expressed genes or domains. The levels of expression of cross-hybridizing sequences were estimated from the Arabidopsis MPSS database to determine if they are particularly highly expressed in roots [26]. The Arabidopsis homologs to cross-hybridizing *Triphysaria *sequences ranged in their root expression between six and over three thousand transcripts per trillion. An ANOVA analysis indicated no significant differences between the predicted expression levels of library specific sequences from false positives present in both forward and subtracted libraries (data not shown).

One possible explanation for the unpredictably large number of false positive clones following SSH procedures is miss-priming at the first or second PCR reactions. cDNAs that were not selected during hybridization would be similarly amplified from both libraries if they have sufficient homology to the 22-mers used in the final amplifications prior to cloning.

### Functional classifications

BLASTX was used to assign putative functions to virtual translations of each library specific assembly. Roughly 75–80% of the library specific sequences had homologies in the *Arabidopsis *protein database at an e value ≤ 10^-8^. Using the AT number of the best *Arabidopsis *hits, the putative *Triphysaria *proteins were placed into functional categories using the Gene Ontology at TAIR [30]. The GO terms obtained for each library are summarized in supplemental table 8. The TAIR output included multiple GO terms for most assemblies so there are more GO descriptors than transcripts.

We used chi squared analyses to determine whether different libraries were enriched for certain GO functional categories (supplemental table 8). The frequency of a particular GO term was determined from the total number of GO terms obtained for that library. The relative frequencies of specific GO terms were then compared between libraries. Table 4 summarizes pair wise comparisons between libraries in the proportion of transcripts in each of the GO categories. The most significant functional enrichment was the overrepresentation of transcripts associated with electron transport in the HF library relative to HR (table 4). Electron transport functions were also enriched in the EDIT library relative to HR although at a lower significance (p ≤ 0.05). Correspondingly, transcripts associated with mitochondria were also over-represented in the HF and EDIT libraries relative to the HR library. The over representation of transcripts associated with electron transport is consistent with the model that haustorial inducing factors trigger development through redox mediated mechanisms [31,32]. The HF library was also enriched for transcripts associated with stress responses. This was previously recognized in the EDIT library and is consistent with the long standing hypothesis that parasitic plants recruit defense related genes for host recognition [7,20].

Transcripts associated with the metabolism of nucleic acids and proteins were significantly less abundant in the HF and EDIT compared to the HR libraries. The down regulation of DNA metabolism genes is consistent with the earlier observations that cell division and DNA synthesis is rapidly terminated in *Striga *upon contact with DMBQ [33]. There were also fewer transcripts predicted to encode protein metabolism functions in the HF and EDIT libraries. While changes in protein profiles have been observed in *Striga *following DMBQ treatment, the overall reduction in the proportion of transcripts encoding protein metabolism genes was not expected [34,35].

## Conclusion

Parasitic plants provide an excellent system for studying genetic mechanisms of chemical signaling between plants. In addition, parasitic weeds are among the world's most destructive agricultural pests against which few genetic resistances are available. Genetic suppression of parasite development at early stages in parasitism is a promising approach for engineering resistance against parasitic weeds but requires knowledge of the genetic factors regulating parasite development. The Pscroph database contains parasitic plant transcripts regulated by host encoded factors; these provide potential points for engineering parasite resistance. More generally, the identification of regulatory elements induced by the presence of other plants provides the potential for genetic weed control strategies.

## Availability and requirements

The Pscroph database can be accessed at .

## Authors' contributions

MJT developed the EST and assembly pipeline and the Pscroph database, AAT and NT made the SSH libraries and prepared them for sequencing, RLR mapped the SSH products to ORFs and manages the Pscroph databases, JIY designed and coordinated the project and wrote the manuscript with input and editing from the other authors. All authors read and approved the final manuscript.

## Supplementary Material

Supplementary files All additional files are in Excel format. Footnote to additional files 1-7: Sequences from three libraries were compared by BLASTN to determine which sequences were present in more than one library. The BLASTN cutoff e value was 10^-80^. Supplemental tables 1-3 contain the library specific sequences and their annotations, tables 4-6 contain sequences present in HF and HR libraries, or HF and EDIT libraries, or HR and EDIT libraries, and table 7 contains sequences present in all three libraries. The annotations shown are parsed from a BLASTX search of the *Arabidopsis *protein database ATH1.pep_cm_20040228.
Footnote to additional file 8: The HF, HR and EDIT specific sequences were annotated with Gene Ontogeny descriptors by cross referencing to the best BLASTX hit in ATH1.pep via TAIR [30]. The first three columns of numbers show the number of times a particular GO descriptor was assigned. Most of the assemblies were annotated with multiple GO descriptors so there are more descriptors than assemblies. A total of 5499 GO descriptors were obtained for the HF library, 7222 GO descriptors from the HR and 8381 descriptors from the EDIT library. Pair-wise comparisons were made between each library for the relative proportion of annotations in a particular GO class. For example, 5499 GO annotations were obtained from the HF assemblies; of these, 666 (12.1%) were annotated as "other physiological processes". Similarly, 931 of the 7222 (12.9%) of the GO annotations obtained from the HR library were "other physiological processes". The resultant Chi-squared value is 1.73, suggesting there are not significant differences in the proportion of assemblies annotated as "other physiological processes" between the HF and HR libraries. In contrast, the relative proportion of "other physiological processes" annotations in the EDIT library is 11.8 % (992 out of 7222 total); this is significantly different from the proportions obtained for the HR library (12.9%) but not the HF (12.1%) at p ≤ 0.05. Numbers in the cells are the Chi-squared values calculated. The yellow shaded cells indicate significant p values ≤ 0.05. Additional file 1 - HF specific assemblies Additional file 2 - HR specific assemblies Additional file 3 - EDIT specific assemblies Additional file 4 - Assemblies in both HF and HR Additional file 5 - Assemblies in both HF and EDIT Additional file 6 - Assemblies in both HR and EDIT Additional file 7 - Assemblies in HF, HR and EDIT Additional file 8 - GO category analysis

## Figures and Tables

**Table 1:** EST and contig statistics of HF, HR and EDIT libraries

	Total ESTs	Assembled Contigs	Assembled Singlets	Total assemblies	% with AT annotation^1^
HF	3386	673	401	1074	82%
HR	3428	781	563	1344	80%
EDIT	2216	403	999	1402	85%

^1 ^AT annotations were determined from BLASTX comparisons with ATH1.pep_cm_20040228 with a cutoff of e ≤ 10^-8^

**Table 2:** Localization of SSH products to gene regions ^1^

Library	Only coding	Only 5' non-coding	Only 3' non-coding	Both 5' and 3' non-coding	Total SSH products analyzed ^2^
HF	1302 (66%)	80 (4%)	566 (29%)	19 (1%)	1967
HR	1212 (54%)	101 (4%)	868 (39%)	51 (2%)	2232
EDIT	721 (38%)	428 (22%)	579 (30%)	171 (9%)	1899

^1^SSH sequences were mapped to the plant gene identified as the best hit in BLASTX searches as described in the text. The number of produces predicted to contain non-coding sequences (3', 5', or both), or only coding sequences, are shown for each library.

^2 ^Redundant ESTs within each library were identified from the BLASTX reports and removed from the analysis. ESTs lacking BLASTX hits with E values ≤ 10^-8 ^were also eliminated from this analysis.

**Table 3:** Occurrence of transcripts in different libraries ^1^

Category	Total # assemblies	# assemblies in category	% category specific
HF specific	1,074	702	65%
HR specific	1,344	910	68%
EDIT specific	1,402	1,018	73%
HF+HR	2,418	213	9%
HF+EDIT	2,476	180	7%
HR+EDIT	2,746	218	8%
HF+HR+EDIT	3,820	92	2%

^1^BLASTN (e ≤ 10^-20^) was used to identify assemblies found in one or more libraries.

**Table 4:** Functional classification of library specific sequences ^1^

Biological function	**HF vs HR**	**HF vs EDIT**	**HR vs EDIT**
cell organization and biogenesis	0.87	0.13	0.43
developmental processes	0.82	0.18	0.31
DNA or RNA metabolism	4.26 *	0.01	4.96 *
electron transport or energy pathways	11.24 ***	2.25	4.33 *
protein metabolism	8.42 **	4.35 *	0.99
response to abiotic or biotic stimulus	2.20	1.31	0.17
response to stress	4.87 *	2.19	0.73
signal transduction	0.27	0.03	0.60
transcription	0.21	0.02	0.12
transport	3.15	0.12	2.51
other biological processes	0.37	0.84	2.80
other cellular processes	1.99	0.05	3.31
other metabolic processes	1.05	0.05	1.93
other physiological processes	1.73	0.24	3.99 *
biological process unknown	0.07	1.58	2.72

^1^The proportion of clones in each library assigned a particular GO function were compared between different libraries and chi square used to indicate significance differences in functional representation between libraries (*** p ≤ 0.001, ** p ≤ 0.01,* p ≤ 0.05).
